# Comparisons Between COVID-19 Stigma and Other Stigmas: Distinct in Explicit Attitudes and Similar in Implicit Process

**DOI:** 10.3389/fpsyg.2022.848993

**Published:** 2022-04-26

**Authors:** Jiajia Zhu, Lihua Yan, Yan Mu

**Affiliations:** ^1^CAS Key Laboratory of Behavioral Science, Institute of Psychology, Chinese Academy of Sciences, Beijing, China; ^2^Department of Psychology, University of Chinese Academy of Sciences, Beijing, China; ^3^Department of Psychological Sciences, University of Connecticut, Storrs, CT, United States

**Keywords:** stigma, pandemic, COVID-19, implicit association test, public stigma

## Abstract

Since the outbreak of COVID-19, the public stigma associated with COVID-19 has emerged. To better understand the COVID-19 stigma, the present research conducted three studies on 1,493 Chinese participants from the outbreak to the recovery period of the COVID-19 pandemic to examine the psychological mechanisms of COVID-19 stigma by comparing it with other disease-related stigmas in terms of their explicit and implicit processes. Study 1 and Study 2 jointly demonstrated that the public endorsed more stigma toward the COVID-19 related people (i.e., the COVID-19 patients) relative to the other disease-related people (i.e., the SARS patients, people with flu) in multiple explicit aspects, including emotional, motivational, cognitive, and social processing. Using the implicit association test (IAT), Study 3 found no significant difference in the implicit measures of the COVID-19 vs. the SARS groups, which further revealed that the pandemic stigmas (i.e., COVID-19 and SARS) were similar at the implicit level. These findings suggest common (implicit level) but distinct (explicit level) psychological processes of the pandemic-related stigmas, which provide reference to policymakers in formulating suitable interventions to deal with COVID-19 stigma and a newly generated potential stigma and provide psychological support for the public in the future.

## Introduction

Since the coronavirus disease 2019 (COVID-19) outbreak, the stigma associated with COVID-19 has been of broad interest to the public, researchers of multiple disciplines (e.g., psychologists, sociologists), and policymakers concerning social harmony and public mental health. A series of studies have investigated the public’s COVID-19 stigma from multiple lenses, including the public’s feelings toward people who are stigmatized because of COVID-19 (e.g., Ransing et al., 2020; Ugidos et al., 2020), people being stigmatized during the pandemic (e.g., Baldassarre et al., 2020; Bruns et al., 2020; Earnshaw et al., 2020; He et al., 2020; Muhidin et al., 2020; Jennings et al., 2021), and their thoughts about the harm of COVID-19 stigma to the public’s health and the whole society (Das, 2020). In addition, the public not only held negative attitudes toward COVID-19 related people and groups but also to the innocent people who were unassociated with COVID-19, such as people with a religious belief (i.e., Muslims) (Islam et al., 2021). The stigma stems from an individual’s high sensitivity and fear of disease infection (Ahorsu et al., 2020). And the COVID-19 related people and groups listed above were stigmatized because of being considered as potential virus carriers (Gronholm et al., 2021). It has been well documented that COVID-19 stigma has caused severe consequences on both the individual and society levels (Das, 2020). At the personal level, experiences of being stigmatized aggravated the psychological problems among people who recovered from COVID-19 and those who have been isolated during the pandemic (Bagcchi, 2020; Xin et al., 2020), hampered the social functioning of stigmatized people (Saeed et al., 2020), and triggered interpersonal conflicts, e.g., being evicting from home (Das, 2020). In addition, COVID-19 stigma as a barrier has been found to impact the effectiveness of pandemic prevention and control (Sotgiu and Dobler, 2020). At the same time, studies have found that COVID-19 stigma could hamper the quality of medical services. During the COVID-19 outbreak, health care workers (HCWs) experienced social stigma against them. This psychological distress could lead to serious psychological outcomes like job burnout, which was extremely detrimental to the prevention and control of the epidemic (Patel et al., 2021). Considering the scope and depth of COVID-19 stigma and its consequences, researchers and policymakers urgently need to acquire knowledge about the characteristics and psychological mechanisms of COVID-19 stigma.

Existing literature on COVID-19 stigma has explored the public’s negative emotions (e.g., fear) toward the COVID-19 patients (Ahorsu et al., 2020; Kumar and Nayar, 2020), avoidance of people residing in the COVID-19 affected regions (Ransing et al., 2020; Xin et al., 2020), and negative attitudes toward people who suffered from COVID-19 (Ugidos et al., 2020). However, most of these studies on COVID-19 stigma assessed only one or two dimensions of COVID-19 stigma and investigated it independently without comparing it with other disease stigmas, which couldn’t address the common and different dimensions of COVID-19 stigma other than other stigmas. Toward a more comprehensive understanding, we aimed to assess multiple dimensions of COVID-19 stigma, including emotional, cognitive, motivational, and social dimensions, and compare it with other disease-related stigmas.

People’s affective responses induced by an emerging infectious disease and their discriminative feelings about people with this disease have attracted growing attention in previous research on stigma. According to the pathogen aversion theory (Park et al., 2003; Peng et al., 2010; Oaten et al., 2011) and the behavioral immune system theory (Schaller and Park, 2011; Murray and Mark, 2016), stigma originates from one’s fear of disease, which in turn cause discrimination and social exclusion toward people who are probably carrying the disease virus. Such negative emotion which reflects an automatic and associative (not cognitive and rule-based) component of encoding the potential dangerousness of the disease-related people has been widely seen in a variety of infectious disease stigmas, such as SARS (Person et al., 2004), Ebola (Overholt et al., 2018) as well as COVID-19 stigma (Taylor et al., 2020). The fear associated with the infection or the quarantine might be the common driver that causes such disease stigmas (Ransing et al., 2020). Fear may be one of the core characteristics of the stigmas associated with infectious diseases compared with those related to other non-infectious diseases. As an infectious disease, COVID-19 has caused tens of millions of confirmed cases and the most deaths worldwide since the 21st century (Liu et al., 2020), we speculated that people might have more fear about people associated with COVID-19 than those related to mild infectious diseases (e.g., flu) and other non-infectious diseases, such as mental illness (e.g., depression).

Accumulating evidence has demonstrated that perceivers, or called stigmatizers, may activate both affective and cognitive processes when they meet and interact with people and groups that are socially stigmatized (Schmidt and Weiner, 1988; Weiner, 1995; Kurzban and Leary, 2001; Pryor et al., 2004; Krendl et al., 2006). For instance, Krendl et al. (2006) using fMRI found that increased activation in the brain areas related to aversive emotions (amygdala and insula) as well as the regions associated with cognitive control (anterior cingulate and lateral prefrontal cortex) were observed when participants were evaluating people from well-established stigmatized groups (e.g., obesity, transsexuality) relative to the controls. Previous research has demonstrated that cognitive attribution (i.e., perceived controllability of disease) could modulate the affective responses of disease stigmas, which help combat the disease and avoid fueling fear (Schmidt and Weiner, 1988; Weiner, 1996). However, little is known about whether the differences between COVID-19 stigma and other stigmas are reflected in the cognitive processes, e.g., cognitive attribution. Previous research has shown that individuals were less likely to attribute the cause of the epidemic disease (i.e., SARS) to the patients than what they did to people with other diseases (i.e., AIDS) (Mak et al., 2006). Accordingly, it is possible that people may endorse less perceived controllability of disease for people and groups related to COVID-19 than those associated with other diseases (e.g., AIDS and mental illness).

From a social psychological perspective, one function of stigmatization is disease avoidance, which hinders social interactions of the stigmatized people (e.g., withdrawing from social situations during COVID-19, Zhang et al., 2020). What’s more, during the COVID-19 outbreak, a wide range of groups, such as healthcare workers, COVID-19 survivors, Asians, were avoided, shunned, or ostracized because they were perceived as the sources of infection (Gronholm et al., 2021). Given the severity and contagion of COVID-19 stigma, it is highly likely that the public would show less approach but more avoidance tendencies for the COVID-19 related group than other disease-related groups during the COVID-19 pandemic.

Another function of public stigma is social norm enforcement (e.g., deviant identity or behavior) (Phelan et al., 2008). The public’s discrimination is viewed as a threat to deviants to conform to the mainstream norms. The stigmatization of deviants protected against infectious diseases, which in turn facilitates survival under pandemic threats (Kurzban and Leary, 2001; Phelan et al., 2008). Considering the public’s intention of norm enforcement during the pandemic, we expected that the public would endorse more deviating from social norms for people and groups related to COVID-19 (i.e., the COVID-19 patients) relative to other mild infectious and common diseases like flu.

While one line of research has been focusing on the explicit processes of stigmatization (i.e., attitudinal, evaluation processing) (Brunstein and Schmitt, 2004), another mainstream of previous research has illuminated the implicit discrimination against the stigmatized groups (i.e., the automatic response based on unconscious processing) (Rydell and McConnell, 2006). People’s implicit attitude is typically assessed by the implicit association test (IAT) – a classical paradigm to measure individual implicit attitudes (Greenwald et al., 2003). Using the IAT, a set of studies have revealed the implicit processing of various disease-related stigmas, such as mental illness (Wang et al., 2012; González-Sanguino et al., 2019) and eating disorders (Elran-Barak et al., 2020) using the IAT paradigm. To be noted, the implicit and explicit components of stigma have proven to be independent (Wang et al., 2012; González-Sanguino et al., 2019). Researchers have demonstrated that people’s implicit attitudes toward others are processed automatically rather than reflectingly in real-time interpersonal communication (Pryora et al., 2013) and could have a greater impact on their actual behavior than explicit attitudes (Kurdi et al., 2019). Sometimes stigmatization could only be observed at the implicit level instead of the explicit level (e.g., González-Sanguino et al., 2019). So far, the implicit processing of COVID-19 stigma was largely neglected, though the public’s explicit attitudes toward people associated with COVID-19 have widely been investigated using a subjective reporting approach. Considering that the importance of the implicit aspect of stigma, we are curious about whether COVID-19 stigma could be detected at the implicit level and whether such stigma may differ from other similar stigma induced by a previous pandemic (i.e., SARS) in terms of its implicit processing.

## The Present Studies

To uncover the implicit and explicit processes underlying COVID-19 stigma, the present research compared participants’ explicit and implicit attitudes toward people associated with COVID-19, we conducted three studies on 1,493 Chinese participants from the outbreak to the recovery period of the COVID-19 pandemic. To test whether and to what extend COVID-19 stigma might be different from a preexisting disease stigma during the COVID-19 outbreak, Study 1 was set out to compare participants’ negative attitudes toward the COVID-19 related group with their attitudes toward the SARS related group and the control group. We selected the SARS-related people as the main control group because the two diseases had similar medical characteristics (e.g., symptoms, the way of transmission) (Saeed et al., 2020). Additionally, Chinese participants have experienced the two infectious diseases. Given that COVID-19 is more severe than SARS in terms of its scope and infectivity, we hypothesized that participants would report more negative attitudes for the COVID-19 related group than the SARS related group and other control groups (i.e., the SARS related people, people from the participants’ permanent residence). To test whether the discrepancy between COVID-19 stigma and SARS stigma remains and which dimensions (e.g., emotional, cognitive) may play a role in differentiating the COVID-19 stigma and other disease stigmas during the recovery stage of COVID-19 when the pandemic has been controlled in China, Study 2 extended single evaluation on negativity to multidimensional measures (i.e., emotional reactions, cognitive processing, withdrawal/approach motivations, social dimensions, and overall evaluations). Additionally, toward a better understanding of the common and distinct aspects underlying COVID-19 stigma, we included various control groups in Study 2, including SARS which is similar to COVID-19, severe infectious diseases (i.e., Ebola and AIDS), the mild and common infectious disease (i.e., cold), non-infectious diseases (e.g., depression and schizophrenia). Based on previous research on disease stigmas (Goffman, 1969; Madru, 2003; Mak et al., 2006; Krendl et al., 2013; Shamblaw et al., 2015; Krendl, 2016; Botha et al., 2017; Das, 2020; Saeed et al., 2020; Taylor et al., 2020; Stevens and Taber, 2021), we hypothesized that participants would report more negative emotions (such as fear), less perceived responsibility of the disease, more avoidance motivation (such as avoidance), and more negative social evaluation (such as social harmfulness) for the COVID-19 related group (vs. other groups we measured). Last but not the least, we were interested in whether the public negative attitudes toward the COVID-19 related people could be internalized and reflected at the implicit level. To address this, by using a revised IAT (Greenwald et al., 2003), Study 3 compared participants’ implicit attitudes (i.e., IAT scores) in the COVID-19 condition relative to those in the preexisting pandemic disease condition (i.e., SARS). If the pandemic-related stigmas shared a common mechanism which is an automatic process (Pryor et al., 2004; Oaten et al., 2011), i.e., instinctive fear of disease, we may expect that there would be no differences in the implicit processing between the two conditions.

## Study 1

### Materials and Methods

#### Participants

Study 1 was conducted online from January 30 to February 3, 2020, in Mainland China, using snowball sampling, which is a widely used method of sampling in qualitative research (Marcus et al., 2017). A sample of 1,179 respondents who passed a probe item was collected. Because of the pandemic severity of Hubei province during the COVID-19 outbreak, we assumed that people from Hubei might have different perceptions of the COVID-19-related people than those from the other provinces did. Therefore, fifty participants from Hubei province and 35 participants who were not in mainland China were not included in the final analysis. Finally, 1,094 participants were included (320 males, 624 females, and 150 participants did not report gender; *Mage* = 34.21 ± 10.39).

#### Method

##### Explicit Stigma

According to previous research (Batson et al., 1997; Corrigan et al., 2015), we measured subjective attitudes toward three target groups on a 7-point scale ranging from 1 = very positive to 7 = very negative, including the COVID-19 group (i.e., the COVID-19 patients, people who have recovered from COVID-19, and people from the high pandemic risk areas), the SARS group (i.e., the SARS patients, and people who have recovered from SARS), and the control group (i.e., people from the participants’ permanent residence).

### Results

#### COVID-19 Stigma at the Outbreak of COVID-19

**FIGURE 1:**
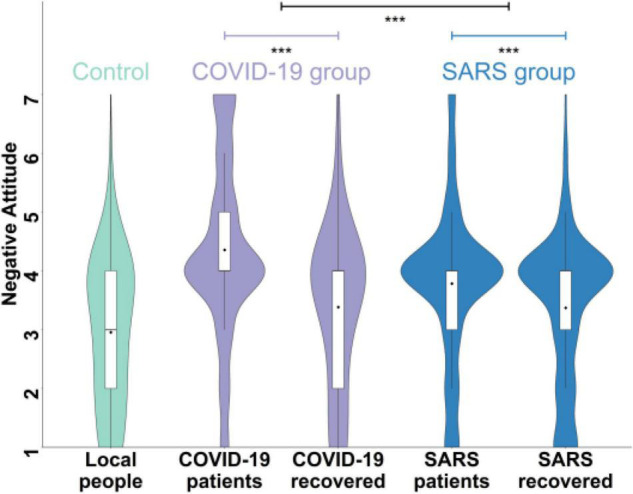
Negative attitudes toward the COVID-19 group (i.e., the COVID-19 patients, people who recovered from COVID-19), the SARS group (i.e., the SARS patients and people who recovered from SARS), and the control group in Study 1. Higher scores indicate more negative attitudes. ***p < 0.001.

Supplementary Table 1 illustrated the negative attitude toward the COVID-19 group and SARS group by gender (33.9% male and 66.1% female) and by age (39.9% younger than 30 years and 60.1% older than 30 years). The results showed that participants’ age and gender did not affect their stigmatization of the COVID-19 group or the SARS group. As predicted, the results of Study 1 showed that participants reported more negative attitudes toward the COVID-19 group relative to the control group [*M*_*COVID*–19_ vs. *M*_*control*_ = 3.87 vs. 2.96, *t*(1087) = 20.35, *p* < 0.001, Cohen’s *d* = 0.62] and the SARS group [*M*_*COVID*–19_ vs. *M*_*SARS*_ = 3.87 vs. 3.58, *t*(1086) = 10.43, *p* < 0.001, Cohen’s *d* = 0.32]. Similarly, participants held more negative attitudes toward the SARS group compared to the control group (*t* = 14.53, *p* < 0.001, Cohen’s *d* = 0.44, Figure 1). These results indicated that the public had discriminative attitudes toward the COVID-19 group and the attitudes of this emerging pandemic were more negative than those of the preexisting disease, i.e., SARS.

Furthermore, we did multiple comparisons between every two items within and between groups (e.g., people who suffered from the diseases vs. people who recovered from the diseases, see Supplementary Table 2 for more details) and found that the discrepancy between COVID-19 and SARS stigma was mainly in people who suffered from the diseases rather than people who recovered from the diseases. To be specific, we conducted a 2 (disease type: COVID-19, SARS) × 2 (target people type: people who suffer from the disease, people who recovered from the disease) repeated measure of ANOVA and found that there was an interaction of disease type and target person type, *F*(1,1086) = 184.85, *p* < 0.001, partial η^2^ = 0.15. *Post hoc* analyses showed that participants’ attitudes toward the COVID-19 patients were more negative than those toward the SARS patients when the target people were patients [*t*(1086) = 15.35, *p* < 0.001]. In contrast, no significant differences were observed in participants’ attitudes toward those recovering from COVID-19 and SARS when the target people were people who have recovered from diseases [*t*(1086) = 0.46, *p*_*tukey*_ = 0.648]. The main effects of disease type [*F* = 108.80, *p* < 0.001, partial η^2^ = 0.09] and the target people type [*F*(1,1086) = 509.35, *p* < 0.001, partial η^2^ = 0.32] were significant.

## Study 2

### Materials and Methods

#### Participants

In Study 2, we recruited 279 participants (102 males, 177 females, *Mage* = 24.71 ± 7.31) from May 2 to July 15, 2020, using an online platform like Qualtrics^1^.

#### Method

##### Explicit Stigma

In addition to the negative evaluation used in Study 1, we included multiple dimensions of stigma in Study 2, including emotional reactions (i.e., fear, sympathy), cognitive processes (i.e., attribution, emotion regulation), withdrawal/approach motivations (i.e., avoidance, helping), and social interactions and evaluations (i.e., becoming neighbors, trust, deviation of social norms, social harmfulness). These measures have been proved to effectively capture different dimensions of stigma (Batson and Ahmad, 2009; Krendl et al., 2012; Shamblaw et al., 2015; Gloor and Puhl, 2016; Botha et al., 2017). Participants scored their probable feelings, reactions, and evaluations about a target person on the 7-point scale ranging from 1 = very unlikely to 7 = very likely (e.g., how likely would you feel fear when you meet a COVID-19 patient). We included: (1) the COVID-19 group, including the COVID-19 patients, people who have recovered from COVID-19, and people from the high pandemic risk areas (i.e., the average score of the evaluations toward people from Hubei and Wuhan, the worst-affected areas in China); (2) the SARS group, including the SARS patients and the people who have recovered from SARS; (3) the other non-pandemic disease groups, including the relatively common disease group (the common flu), the severe infectious diseases group (i.e., AIDS, Ebola), and the mental disease group (i.e., depression, and schizophrenia). The disease groups listed above have been widely studied in previous studies (e.g., Thornicroft et al., 2009; Nyblade et al., 2018; Javed et al., 2021; Reinius et al., 2021). We included the non-disease healthy control (i.e., healthy people), the religious group (i.e., Muslims), and the moral violation group (i.e., robbers) as the non-disease stigmatized controls (Lai and Kao, 2018; Islam et al., 2021).

### Results

#### COVID-19 Stigma at the Recovery Period of COVID-19

Consistent with Study 1, Study 2 showed that participants’ age (90.0% younger than 30 years and 10.0% older than 30 years) and gender (38.4% male and 66.7% female) did not affect their stigmatization of the COVID-19 group or the SARS group (see Supplementary Table 3).

Consistent with Study 1, Study 2 conducted during the recovery period of COVID-19 replicated that participants reported more negative attitudes toward the COVID-19 group than the control group [*M*_*COVID*–19_ vs. *M*_*control*_ = 4.05 vs. 2.14, *t*(278) = 20.90, *p* < 0.001, Cohen’s *d* = 1.25] and the SARS group [*M*_*COVID*–19_ vs. *M*_*SARS*_ = 4.05 vs. 3.93, *t*(278) = 2.39, *p* = 0.017, Cohen’s *d* = 0.14]. Participants held more negative attitudes toward the SARS group compared to the control group [*t*(278) = 19.96, *p* < 0.001, Cohen’s *d* = 1.20, Supplementary Figure 1].

Similarly, the 2 (disease type: COVID-19, SARS) × 2 (target people type: patients and people recovering from the disease) ANOVA replicated that participants held more negative attitudes toward the COVID-19 patients relative to the SARS patients [*t*(278) = 3.23, *p* < 0.001] and no difference was observed when the target were people who recovered from the diseases [*t*(278) = 0.11, *p* = 0.909]. The results of Study 1 and 2 consistently demonstrated that COVID-19 stigma is more severe than SARS stigma and the more discriminative attitudes toward the COVID-19 relative to the SARS group were mainly reflected in how they perceive and evaluate the COVID-19 (vs. SARS) patients.

**FIGURE 2:**
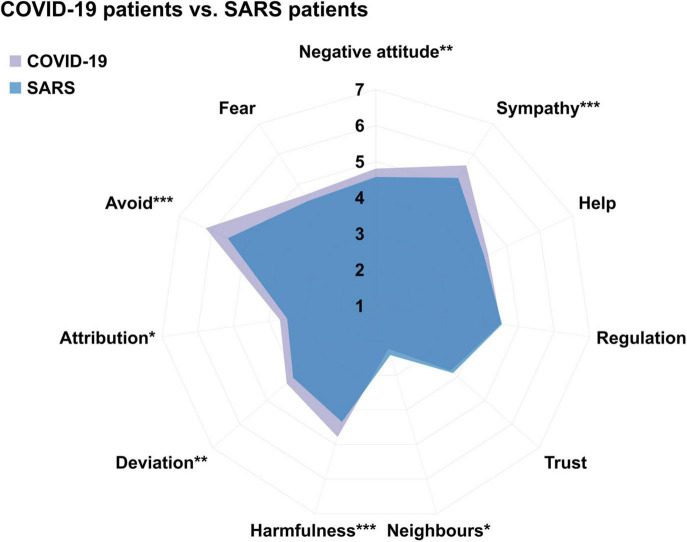
Participants’ explicit attitudes toward the COVID-19 patients versus the SARS patients in terms of emotional (i.e., fear and sympathy), cognitive (i.e., attribution and regulation), motivational (i.e., avoid, help, and the willingness to become neighbors), and social dimensions (i.e., social harmfulness, social deviant, and trust) in Study 2. Higher score indicates a higher level of possibility. ^∗^*p* < 0.05, ^∗∗^*p* < 0.01, ^∗∗∗^*p* < 0.001.

To further illuminate what may drive the public’s negative attitudes toward the COVID-19 patients (vs. the SARS patients), we compared their attitudes for the two groups in multiple dimensions, including emotional, cognitive, motivational, and social dimensions (Figure 2). The results showed that participants reported no difference in the possibility of feeling fear of the COVID-19 vs. the SARS patients [*M*_*COVID–*19_ vs. *M*_*SARS*_ = 4.66 vs. 4.47, *t*(278) = 1.76, *p* = 0.080, Cohen’s *d* = 0.11], but were more likely to have sympathy for the COVID-19 vs. the SARS patients [*M*_*COVID–*19_ vs. *M*_*SARS*_ = 5.63 vs. 5.22, *t*(278) = 4.93, *p* < 0.001, Cohen’s *d* = 30]. As for the cognitive dimension, they reported higher possibility of attributing the cause of the disease to the COVID-19 than the SARS patients [*M*_*COVID–19*_ vs. *M*_*SARS*_ = 3.69 vs. 3.49, *t*(278) = 2.36, *p* = 0.019, Cohen’s *d* = 0.14], but held the same degree of emotion regulation when they met the COVID-19 patients relative to the SARS patients [*M*_*COVID–*19_ vs. *M*_*SARS*_ = 4.50 vs. 4.53, *t*(278) = −0.33, *p* = 0.738, Cohen’s *d* = −0.02]. At the motivational aspect, participants rated higher possibility of keeping avoidance of the COVID-19 patients (vs. the SARS patients) [*M*_*COVID–19*_ vs. *M*_*SARS*_ = 6.19 vs. 5.52, *t*(278) = 6.71, *p* < 0.001, Cohen’s *d* = 0.40], but rated the same level of willingness to help the COVID-19 and the SARS patients [*M*_*COVID–19*_ vs. *M*_*SARS*_ = 4.43 vs. 4.30, *t*(278) = 1.59, *p* = 0.113, Cohen’s *d* = 0.10]. As for the social evaluation, participants endorsed more social norm violations [*M*_*COVID–19*_ vs. *M*_*SARS*_ = 4.28 vs. 4.04 *t*(278) = 2.65, *p* = 0.009, Cohen’s *d* = 0.16] and a higher level of social harmfulness for the COVID-19 than the SARS patients [*M*_*COVID–19*_ vs. *M*_*SARS*_ = 4.78 vs. 4.35, *t*(278) = 5.21, *p* < 0.001, Cohen’s *d* = 0.31]. Meanwhile, participants reported lower possibility of becoming neighbors with the COVID-19 patients compared to the SARS patients [*M*_*COVID–19*_ vs. *M*_*SARS*_ = 2.24 vs. 2.42, *t*(278) = −2.03, *p* = 0.043, Cohen’s *d* = −0.12].

Consistently, similar differences in the motivational, cognitive, and social dimensions were observed in participants’ attitudes toward people who recovered from COVID-19 vs. people who recovered from SARS (Supplementary Figure 2). Similarly, the results showed that participants reported more sympathetic feelings of, more withdrawal tendencies for, and less willingness of being neighbors with the COVID-19 patients relative to the other disease patients (i.e., SARS, Ebola, AIDS, and flu) (Supplementary Figure 3). It is noteworthy that participants had the highest possibility of avoidance tendency for the COVID-19 patients among all the groups we measured.

In summary, these results suggest that the public’s COVID-19 stigma is different from a set of severe and mild infectious disease stigmas (i.e., AIDS, SARS, Ebola, and flu) at multiple explicit aspects, including emotional, cognitive, motivational, and social dimensions.

## Study 3

### Materials and Methods

#### Participants

In Study 3, we first calculated the statistical effect size by G*power 3.1 using a prior analysis (Faul et al., 2007). To achieve the effect size of 0.8 (α = 0.05, two-tailed) in the paired sample *t*-test, the sample size recommended by G*power was 34. We recruited 35 Chinese participants (12 males, 23 females, *Mage* = 24.03 ± 4.08) from November 10 to December 10, 2020. The informed consent of all participants in the three studies was obtained before the studies. The research was approved by the Local Research Ethics Committee.

#### Method

##### Implicit Stigma

We adopted the standard seven-step IAT to measure the implicit attitude of COVID-19 stigma (Greenwald et al., 2003). Experimental materials were determined by a pilot study in which 30 participants were asked to write a list of words that could describe COVID-19. We then selected the top six words with the highest frequency (e.g., the COVID-19 patients, the suspected COVID-19 patients, the people who carry the COVID-19 virus) as the final stimuli for the COVID-19 condition. For the SARS condition, we replace the word “COVID-19” with “SARS.” For the control group, we used people without diseases as stimuli (e.g., ordinary people, members of society, healthy people). Consistent with previous research, we included the positive (i.e., amiable, approachable, non-contagious, harmless, non-threatening, positive) and negative adjectives (i.e., scary, aloof, contagious, harmful, threatening, negative) as the attribute words (Greenwald et al., 1998). The experimental procedure was compiled by PsychoPy 3 (Peirce, 2007). During the experiment, category labels (i.e., COVID-19/SARS/Control) and attribute words (i.e., positive/negative attribute words) were displayed on the screen’s upper left and right corners, respectively, while stimuli (i.e., words of the COVID-19/SARS/control group) were displayed in the center of the screen. The order of the COVID-19 and SARS conditions was balanced among participants.

##### Explicit Stigma

Similar to Study 2, we assessed explicit aspects of COVID-19 and SARS stigmas on a 7-point scale ranging from 1 (very positive) to 7 (very negative).

### Results

#### Implicit Attitudes of COVID-19 Stigma

First, at the explicit levels, the results of Study 3 showed that participants reported more negative attitudes toward the COVID-19 group than the SARS group [*M*_*COVID*–19_ vs. *M*_*SARS*_ = 4.06 vs. 3.81, *t*(27) = 2.10, *p* = 0.045, Cohen’s *d* = 0.41] and the control group [*M*_*COVID*–19_ vs. *M*_*control*_ = 4.06 vs. 2.26, *t*(27) = 6.53, *p* < 0.001, Cohen’s *d* = 1.26], which was consistent with the results of Study 1 and 2 (Supplementary Figure 4).

**FIGURE 3:**
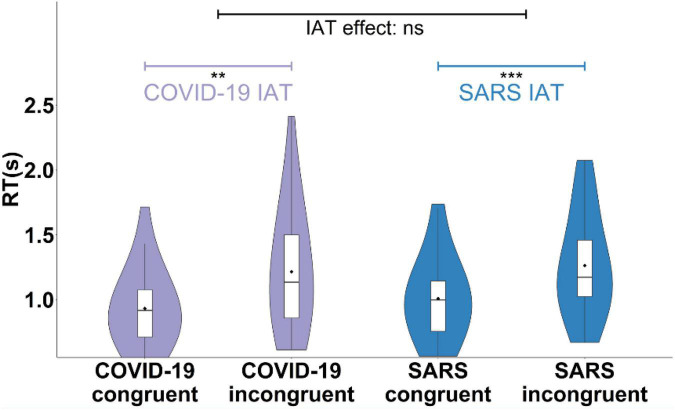
Implicit attitudes of the COVID-19 group and the SARS group. The reaction times (RT) of the incongruent and congruent trials in the COVID-19 and SARS conditions. **p < 0.01, ***p < 0.001.

At the implicit level, the results of IAT showed that the reaction times (RTs) of the incongruent trials were longer than those of the congruent trials across different conditions (Figure 3). Consistent with previous research (Greenwald et al., 2003), we computed the most common score for the IAT (D score) for each condition to conduct further analyses. The results showed that *D_*COVID–*19_* (0.43, 95% *CI* = [0.30, 0.57]) and *D*_*SARS*_ (0.36, 95% *CI* = [0.23, 0.49]) were both significant, indicating that there were implicit negative attitudes toward people associated with COVID-19 and SARS. No significant difference was found in the D scores of the COVID-19 vs. the SARS conditions [*t*(31) = 1.24,*p* = 0.22, Cohen’s *d* = 0.22]. There was no correlation between participants’ explicit and implicit attitudes toward the COVID-19 group [*r*(27) = 0.25, *p* = 0.22], suggesting that the two attitudes may be independent of each other.

## Discussion

COVID-19 stigma poses a severe threat to the mental health and social function of a wide range of people (i.e., people who have recovered from COVID-19) and groups (i.e., Asian, Chinese) (Bavel et al., 2020; Zhao et al., 2021). Many efforts have been made to understand and deal with such stigma. For example, WHO (World Health Organization) suggested that social media needed to carefully use words when describing this disease to prevent and reduce stigmatization at the beginning of the COVID-19 outbreak (World Health Organization [WHO], 2020). In addition, psychologists have attempted to uncover the psychological mechanisms underlying the disease-related stigmas and search for the possible way of individual and group intervention (Pappas et al., 2009; Morris, 2012; White Hughto et al., 2015; Fischer et al., 2019; Stangl et al., 2019). Along this line of research, the first study collected 1,094 Chinese participants from January to February 2020 online and revealed that the public endorsed more negative attitudes toward the COVID-19 related people (i.e., the COVID-19 patients, people who have recovered from COVID-19, and people from the high pandemic risk areas) than the SARS related people (i.e., the SARS patients, and people who have recovered from SARS) and the control group (i.e., people from the participants’ permanent residence) at the early stage of the COVID-19 pandemic. The second study further revealed that COVID-19 stigma remained at the late stage of COVID-19 and was more severe than other preexisting stigmas in multiple aspects, including the higher possibility of attributing the cause of the disease to the person, more keeping avoidance of, less willingness of becoming neighbors, higher levels of social harmfulness, and more deviating from social norms. Furthermore, using the IAT tasks, the third study further demonstrated that the implicit COVID-19 stigma existed at the late stage of COVID-19 and no significant difference was shown in the implicit processing between COVID-19 and SARS stigma. Taken together, the current research provided the first empirical evidence for the explicit and implicit aspects of COVID-19 stigma and its common and unique processes with other stigmas.

Prior studies on the pandemic stigmas have uncovered the public’s discriminatory attitudes toward disease-related people and groups (Ahorsu et al., 2020; Kumar and Nayar, 2020; Ransing et al., 2020; Ugidos et al., 2020; Xin et al., 2020; Nguyen et al., 2021). Among previous studies, a few attempts have been made to compare the newly emerging pandemic disease-related stigma with other stigmas. For example, Des Jarlais et al. (2006) found no difference between United States participants’ attitudes toward AIDS and SARS. The combined fear of the disease and the sufferers led to similar patterns of the two stigmas, such as social exclusion (Des Jarlais et al., 2006). Similarly, Li M. et al. (2021) compared COVID-19 stigma with AIDS stigma during the outbreak of COVID-19 and found that Chinese participants had a higher level of avoidance for the COVID-19 patients relative to the AIDS patients. Though these studies compared the COVID-19 stigma with the existing disease stigma, namely AIDS, they may have some limitations. For instance, AIDS differs from the two pandemic diseases (i.e., SARS and COVID-19) in many aspects, such as symptoms, curability, and the way of transmission (Li M. et al., 2021). Some researchers have pointed out that the stigma associated with different pandemics may have different characteristics and that such differences may be caused by differences in the disease (e.g., the way of transmission) (Saeed et al., 2020). Considering the similarity between COVID-19 and SARS in terms of pathogenesis and clinical features (Caldaria et al., 2020), Study 1 using the common control and SARS group as controls first demonstrated that participants of different ages and genders consistently reported more negative attitudes toward the COVID-19 group (vs. the SARS group) at the outbreak and recovery period of COVID-19. Specifically, this difference was mainly reflected in the participants’ negative evaluation of patients with the disease rather than those who recovered from the disease. One possible account could be that the public may perceive more risks of COVID-19 than SARS due to the fact that the former is more severe and threatens their present life but the latter has receded for nearly 20 years. In addition, our data showed that the difference between the public’s negative attitudes toward the COVID-19 patients and those for the SARS patients difference tended to narrow as the COVID-19 pandemic entered into the recovery stage. Similarly, Li Q. et al. (2021) found that when a pandemic disease broke out, the perception of uncertainty about the pandemic environment may increase the severity of stigma. Consistent with the previous work, our results suggest that when facing with a new pandemic disease, effective pandemic prevention and control can not only effectively protect people’s physical health (Kang et al., 2020; Gelfand et al., 2021) but also reduce the pandemic disease-related stigma to promote interpersonal collaboration.

To be noted, our study is the first to compare COVID-19 stigma with other disease-related stigmas at multiple dimensions, including emotional responses, cognitive processes, motivational, and social evaluations. On the one hand, our results suggest that there might be a common mechanism among disease-related stigmas. Specifically, the common drive is highly likely to be reflected in the emotional dimension, i.e., fear. Such negative emotion has been widely discussed in previous research (Ransing et al., 2020). Previous studies have shown that fear occurs in the early stages of disease-related stigma processing, which may involve an automated process (Pryor et al., 2004; Oaten et al., 2011). In the process of further cognitive processing, motivation tendency, and social evaluation, we found that participants reported more withdrawal tendencies for, and less willingness of being neighbors with the COVID-19 patients relative to the other disease patients (i.e., SARS, Ebola, AIDS, and flu). Future research may explore at which stage of the stigma processing the more significant stigma of the newly generated pandemic disease occurs and explore the psychological processes involved in this process.

On the other hand, our study suggested that COVID-19 stigma and other disease stigmas differ in multiple dimensions. For example, participants’ negative attitudes toward the COVID-19 patients were more significant than their negative attitudes toward the SARS patients but less than their negative attitudes toward the AIDS and Ebola patients. Similarly, in the social evaluation and cognitive processing dimensions, participants stigmatized the COVID-19 patients (e.g., social deviant, social harmfulness, and attribution) more than the SARS patients and the patients with mild infectious diseases (i.e., the common flu), but less than the severe infectious diseases (i.e., AIDS and Ebola) and the psychiatric disorders (i.e., schizophrenia and depression). The above results collectively indicate that the public’s COVID-19 stigma is more severe than the stigmas related to most of the existing infectious diseases but less than those associated with severe infectious diseases and mental illnesses in the cognitive processing and social evaluations (Supplementary Figure 3). In the motivation dimension, participants reported the highest avoidance for the COVID-19 patients (vs. the patients with all other diseases we measured). In the negative emotional dimension, COVID-19 stigma, as a category of infectious disease-related stigma, had no significant difference in the emotion dimension (i.e., fear) compared with other infectious disease stigmas. However, compared with non-communicable diseases (e.g., mental illness), participants had a higher degree of fear of the COVID-19-related group. This may indicate that the emotion help distinguish infectious disease stigma from non-communicable disease stigma. However, in the positive emotional dimension (i.e., sympathy), we found that participants reported the highest possibility of feeling sympathy for the COVID-19 patients (vs. the patients with all other diseases we measured). Although studies have documented a negative association between compassion and stigma (Batson et al., 1997; Galinsky and Moskowitz, 2000; Stark et al., 2013; Boag and Carnelley, 2016; Álvarez-Castillo et al., 2018; van Bommel et al., 2021). The public’s sympathy for people associated with the pandemic disease might be crucial to the development of stigmatization interventions in future research. In sum, our research, using a horizontal comparison among the newly emerging pandemic stigma and other stigmas, is conducive to precisely characterizing and differentiating the pandemic-related stigma (vs. the other disease stigma).

The IAT results demonstrated that the implicit COVID-19 stigma existed and further revealed the separation of explicit and implicit stigma toward COVID-19-related people. Our findings contribute to this field by providing first evidence on a common implicit basis for processing pandemic stigmas. Additionally, we found no differences in the public’s implicit attitudes toward the newly emerging pandemic disease (i.e., COVID-19) and the existing pandemic disease (i.e., SARS). Implicit stigma reflects the connection between the target people and negative characteristics in the early stage of individual cognitive processing (Greenwald et al., 2003), which is linked with emotional processing (Pryor et al., 2004; Oaten et al., 2011). This is consistent with the results of our research that the public has the same degree of fear for the COVID-19 group and the SARS group. This suggests that there may be consistent activities in the neural processing of pandemic disease-related stigma, such as the activities of the amygdala and insula (Harris and Fiske, 2006; Krendl et al., 2006, 2013; Krendl and Cassidy, 2017; Finnell, 2018). Combined with our multidimensional exploration of the stigma of different diseases, future research needs to explore how the public stigma of different diseases is differentiated from the same processing, what neural or psychological activities are involved in this process, and how it is affected by the social environment.

### Limitations and Future Research

The first limitation of our study was that Study 3 testing the COVID-19 implicit stigma was conducted during the recovery period of the pandemic in China. Thus, whether the implicit COVID-19 stigma might exist at the outbreak period of a new pandemic remains an open question. Another shortcoming is that longitudinal design may be more suitable for addressing the dynamic changes of explicit and implicit COVID-19 stigma over time. Additionally, we only tested our hypothesis in Chinese participants. Future cross-cultural work is suggested to examine the cultural variations on such stigma. Using fMRI, future research could address the cultural differences in the neural activity of processing pandemic-related stigmas. Moreover, previous studies have shown that implicit attitudes are relatively stable and difficult to change (Gawronski et al., 2017; Payne et al., 2017; Vuletich and Payne, 2019). Therefore, how to modulate the deep-rooted implicit stigma is still an open question that future research can explore. From a research perspective, the current research mainly has focused on the public point of view on COVID-19 stigma. To be noted, there is another important perspective from the stigmatized people and groups—self-stigma or internalized stigma (Mak et al., 2007; Wu et al., 2015). Future research can explore the relationship between the COVID-19- related public stigma and self-stigma to have a more comprehensive understanding of pandemic-related stigma.

## Conclusion

The current results shed new light on the newly emerging pandemic stigma which involves in common (implicit level) but distinct (explicit level) psychological processes. The present findings enrich the existing literature on the mechanism underlying pandemic-related stigma. Future research is encouraged to step further on the neural mechanisms underlying such stigma. For policymakers, developing effective disease prevention and control policies and reducing infection risks in the environment are key to promoting stable social functioning. At the same time, because the public’s stigma toward the COVID-19 patients is mainly manifested in their avoiding motivation, policymakers and health workers should strive to ensure that reasonable social distancing can be maintained in public places to eliminate the uncertain risk of the disease and thereby reduce the stigma.

## Data Availability Statement

De-identified data and code of the present study are available upon request to the corresponding author.

## Ethics Statement

The studies involving human participants were reviewed and approved by the Ethics Committee of the Institute of Psychology, Chinese Academy of Sciences. The participants provided their written informed consent to participate in this study.

## Author Contributions

YM conceived the project. YM and JZ designed the project and wrote the manuscript. JZ and LY implemented the experiment and collected the data. JZ pre-processed the data and performed the analyses. All authors discussed the results.

## Conflict of Interest

The authors declare that the research was conducted in the absence of any commercial or financial relationships that could be construed as a potential conflict of interest.

## Publisher’s Note

All claims expressed in this article are solely those of the authors and do not necessarily represent those of their affiliated organizations, or those of the publisher, the editors and the reviewers. Any product that may be evaluated in this article, or claim that may be made by its manufacturer, is not guaranteed or endorsed by the publisher.

## Footnotes

1
https://www.wenjuan.com

## References

[B1] Ahorsu D. K. Lin C. Y. Imani V. Saffari M. Griffiths M. D. Pakpour A. H. (2020). The fear of COVID-19 scale: development and initial validation. *Int. J. Ment. Health Addict.* [Epub ahead of print]. 10.1007/s11469-020-00270-8 32226353 PMC7100496

[B2] Álvarez-Castillo J. L. Fernández-Caminero G. González-González H. (2018). Is empathy one of the big three? Identifying its role in a dual-process model of ideology and blatant and subtle prejudice. *PLoS One* 13:e0195470. 10.1371/JOURNAL.PONE.0195470 29621307 PMC5886567

[B3] Bagcchi S. (2020). Stigma during the COVID-19 pandemic. *Lancet Infect. Dis.* 20:782. 10.1016/S1473-3099(20)30498-9 32592670 PMC7314449

[B4] Baldassarre A. Giorgi G. Alessio F. Lulli L. G. Arcangeli G. Mucci N. (2020). Stigma and discrimination (Sad) at the time of the sars-cov-2 pandemic. *Int. J. Environ. Res. Public Health* 17:6341. 10.3390/ijerph17176341 32878180 PMC7503800

[B5] Batson C. D. Ahmad N. Y. (2009). Using empathy to improve intergroup attitudes and relations. *Soc. Issues Policy Rev.* 3 141–177. 10.1111/j.1751-2409.2009.01013.x

[B6] Batson C. D. Polycarpou M. P. Harmon-Jones E. Imhoff H. J. Mitchener E. C. Bednar L. L. (1997). Empathy and attitudes: can feeling for a member of a stigmatized group improve feelings toward the group? *J. Pers. Soc. Psychol.* 72 105–118. 10.1037//0022-3514.72.1.105 9008376

[B7] Bavel J. J. V. Baicker K. Boggio P. S. Capraro V. Cichocka A. Cikara M. (2020). Using social and behavioural science to support COVID-19 pandemic response. *Nat. Hum. Behav.* 4 460–471. 10.1038/s41562-020-0884-z 32355299

[B8] Boag E. M. Carnelley K. B. (2016). Attachment and prejudice: the mediating role of empathy. *Br. J. Soc. Psychol.* 55 337–356. 10.1111/BJSO.12132 26549740

[B9] Botha F. B. Shamblaw A. L. Dozois D. J. A. (2017). Reducing the stigma of depression among Asian students: a social norm approach. *J. Cross Cult. Psychol.* 48 113–131. 10.1177/0022022116674598

[B10] Bruns D. P. Kraguljac N. V. Bruns T. R. (2020). COVID-19: facts, cultural considerations, and risk of stigmatization. *J. Transcult. Nurs.* 31 326–332. 10.1177/1043659620917724 32316872 PMC7324134

[B11] Brunstein J. C. Schmitt C. H. (2004). Assessing individual differences in achievement motivation with the Implicit Association Test. *J. Res. Pers.* 38 536–555. 10.1016/j.jrp.2004.01.003

[B12] Caldaria A. Conforti C. Di Meo N. Dianzani C. Jafferany M. Lotti T. (2020). COVID-19 and SARS: differences and similarities. *Dermatol. Ther.* 33:e13395. 10.1111/dth.13395 32277530 PMC7235519

[B13] Corrigan P. W. Bink A. B. Fokuo J. K. Schmidt A. (2015). The public stigma of mental illness means a difference between you and me. *Psychiatry Res.* 226 186–191. 10.1016/j.psychres.2014.12.047 25660735

[B14] Das M. (2020). Social construction of stigma and its implications–observations from COVID-19. *SSRN Electron. J.* Available online at: https://ssrn.com/abstract=3599764 (Preprint).

[B15] Des Jarlais D. C. Galea S. Tracy M. Tross S. Vlahov D. (2006). Stigmatization of newly emerging infectious diseases: AIDS and SARS. *Am. J. Public Health* 96 561–567. 10.2105/AJPH.2004.054742 16449597 PMC1470501

[B16] Earnshaw V. A. Brousseau N. M. Hill E. C. Kalichman S. C. Eaton L. A. Fox A. B. (2020). Anticipated stigma, stereotypes, and COVID-19 testing. *Stigma Health* 5 390–393. 10.1037/sah0000255

[B17] Elran-Barak R. Dror T. Goldschmidt A. B. Teachman B. A. (2020). The implicit association of high-fat food and shame among women recovered from eating disorders. *Front. Psychol.* 11:1068. 10.3389/fpsyg.2020.01068 32581937 PMC7283547

[B18] Faul F. Erdfelder E. Lang A.-G. Axel B. (2007). G* power 3: a flexible statistical power analysis program for the social, behavioral, and biomedical sciences. *Behav. Res. Methods* 39 175–191. 10.3758/BF03193146 17695343

[B19] Finnell D. S. (2018). A call to action: managing the neural pathway of disgust, bias, prejudice, and discrimination that fuels stigma. *Subst. Abuse* 39 399–403. 10.1080/08897077.2019.1576091 30901305

[B20] Fischer L. S. Mansergh G. Lynch J. Santibanez S. (2019). Addressing disease-related stigma during infectious disease outbreaks. *Disaster Med. Public Health Prep.* 13 989–994. 10.1017/dmp.2018.157 31156079 PMC6889068

[B21] Galinsky A. D. Moskowitz G. B. (2000). Perspective-taking: decreasing stereotype expression, stereotype accessibility, and in-group favoritism. *J. Pers. Soc. Psychol.* 78 708–724. 10.1037/0022-3514.78.4.708 10794375

[B22] Gawronski B. Morrison M. Phills C. E. Galdi S. (2017). Temporal stability of implicit and explicit measures: a longitudinal analysis. *Pers. Soc. Psychol. Bull.* 43 300–312. 10.1177/0146167216684131 28903689

[B23] Gelfand M. J. Jackson J. C. Pan X. Nau D. Pieper D. Denison E. (2021). The relationship between cultural tightness–looseness and COVID-19 cases and deaths: a global analysis. *Lancet Planet. Health* 5 e135–e144. 10.1016/S2542-5196(20)30301-6 33524310 PMC7946418

[B24] Gloor J. L. Puhl R. M. (2016). Empathy and perspective-taking: examination and comparison of strategies to reduce weight stigma. *Stigma Health* 1 269–279. 10.1037/sah0000030

[B25] Goffman E. (1969). Stigma: notes on the management of spoiled identity. *Postgrad. Med. J.* 45 642–642. 10.2307/2575995

[B26] González-Sanguino C. Muñoz M. Castellanos M. A. Pérez-Santos E. Orihuela-Villameriel T. (2019). Study of the relationship between implicit and explicit stigmas associated with mental illness. *Psychiatry Res.* 272 663–668. 10.1016/j.psychres.2018.12.172 30616138

[B27] Greenwald A. G. McGhee D. E. Schwartz J. L. (1998). Measuring individual differences in implicit cognition: the implicit association test. *J. Pers. Soc. Psychol.* 74, 1464–1480. 10.1037/0022-3514.74.6.1464 9654756

[B28] Greenwald A. G. Nosek B. A. Banaji M. R. (2003). Understanding and using the implicit association test: I. An improved scoring algorithm. *J. Pers. Soc. Psychol.* 82 197–216. 10.1037/0022-3514.85.2.197 12916565

[B29] Gronholm P. C. Nose M. Van Brakel W. H. Eaton J. Ebenso B. Fiekert K. (2021). Reducing stigma and discrimination associated with COVID-19: early stage pandemic rapid review and practical recommendations. *Epidemiol. Psychiatr. Sci.* 30:e15. 10.1017/S2045796021000056 33504412 PMC7884669

[B30] Harris L. T. Fiske S. T. (2006). Dehumanizing the lowest of the low: neuroimaging responses to extreme out-groups. *Psychol. Sci.* 17 847–853. 10.4324/9781315187280-8 17100784

[B31] He J. He L. Zhou W. Nie X. He M. (2020). Discrimination and social exclusion in the outbreak of COVID-19. *Int. J. Environ. Res. Public Health* 17 17–20. 10.3390/ijerph17082933 32340349 PMC7215298

[B32] Islam A. Pakrashi D. Vlassopoulos M. Choon L. (2021). Stigma and misconceptions in the time of the COVID-19 pandemic?: a field experiment in India. *Soc. Sci. Med.* 278:113966. 10.1016/j.socscimed.2021.113966 33940435 PMC8080503

[B33] Javed A. Lee C. Zakaria H. Buenaventura R. D. Cetkovich-Bakmas M. Duailibi K. (2021). Reducing the stigma of mental health disorders with a focus on low- and middle-income countries. *Asian J. Psychiatry* 58:102601. 10.1016/j.ajp.2021.102601 33611083

[B34] Jennings W. Stoker G. Valgarğsson V. Devine D. Gaskell J. (2021). How trust, mistrust and distrust shape the governance of the COVID-19 crisis. *J. Eur. Public Policy* 28 1174–1196. 10.1080/13501763.2021.1942151

[B35] Kang C. Meng F. Feng Q. Yuan J. Liu L. Xu L. (2020). Implementation of quarantine in China during the outbreak of COVID-19. *Psychiatry Res.* 289:113038. 10.1016/j.psychres.2020.113038 PMC7190468 32387796

[B36] Krendl A. C. (2016). An fMRI investigation of the effects of culture on evaluations of stigmatized individuals. *Neuroimage* 124 336–349. 10.1016/j.neuroimage.2015.08.030 26302670

[B37] Krendl A. C. Cassidy B. S. (2017). Neural response to evaluating depression predicts perceivers’ mental health treatment recommendations. *Cogn. Affect. Behav. Neurosci.* 17 1084–1097. 10.3758/s13415-017-0534-8 28895092 PMC5806511

[B38] Krendl A. C. Kensinger E. A. Ambady N. (2012). How does the brain regulate negative bias to stigma? *Soc. Cogn. Affect. Neurosci.* 7 715–726. 10.1093/scan/nsr046 21896496 PMC3427867

[B39] Krendl A. C. Macrae C. N. Kelley W. M. Fugelsang J. A. Heatherton T. F. (2006). The good, the bad, and the ugly: an fMRI investigation of the functional anatomic correlates of stigma. *Soc. Neurosci.* 1 5–15. 10.1080/17470910600670579 18633772

[B40] Krendl A. C. Moran J. M. Ambady N. (2013). Does context matter in evaluations of stigmatized individuals? An fMRI study. *Soc. Cogn. Affect. Neurosci.* 8 602–608. 10.1093/scan/nss037 22451481 PMC3682444

[B41] Kumar A. Nayar K. R. (2020). COVID-19: stigma, discrimination, and the blame game. *Int. J. Ment. Health* 49 382–384. 10.1080/00207411.2020.1809935

[B42] Kurdi B. Mann T. C. Charlesworth T. E. S. Banaji M. R. (2019). The relationship between implicit intergroup attitudes and beliefs. *Proc. Natl. Acad. Sci. U.S.A.* 116 5862–5871. 10.1073/pnas.1820240116 30833402 PMC6442597

[B43] Kurzban R. Leary M. R. (2001). Evolutionary origins of stigmatization: the functions of social exclusion. *Psychol. Bull.* 127 187–208. 10.1037/0033-2909.127.2.187 11316010

[B44] Lai T. Kao G. (2018). Hit, robbed, and put down (but not bullied): underreporting of bullying by minority and male students. *J. Youth Adolesc.* 47 619–635. 10.1007/s10964-017-0748-7 28929272

[B45] Li M. Long J. Wang X. Liao Y. Liu Y. Hao Y. (2021). A comparison of COVID-19 stigma and AIDS stigma during the COVID-19 pandemic: a cross-sectional study in China. *Front. Psychiatry* 12:782501. 10.3389/fpsyt.2021.782501 34925108 PMC8671734

[B46] Li Q. Luo R. Zhang X. Meng G. Dai B. Liu X. (2021). Intolerance of covid-19-related uncertainty and negative emotions among Chinese adolescents: a moderated mediation model of risk perception, social exclusion and perceived efficacy. *Int. J. Environ. Res. Public Health* 18:2864. 10.3390/ijerph18062864 33799731 PMC8002157

[B47] Liu Y. C. Kuo R. L. Shih S. R. (2020). COVID-19: the first documented coronavirus pandemic in history. *Biomed. J.* 43 328–333. 10.1016/j.bj.2020.04.007 32387617 PMC7199674

[B48] Madru N. (2003). Stigma and HIV: does the social response affect the natural course of the epidemic? *J. Assoc. Nurses AIDS Care* 14 39–48. 10.1177/1055329003255112 14571685

[B49] Mak W. W. S. Mo P. K. H. Cheung R. Y. M. Woo J. Cheung F. M. Lee D. (2006). Comparative stigma of HIV/AIDS, SARS, and Tuberculosis in Hong Kong. *Soc. Sci. Med.* 63 1912–1922. 10.1016/j.socscimed.2006.04.016 16766106 PMC7115765

[B50] Mak W. W. S. Poon C. Y. M. Pun L. Y. K. Cheung S. F. (2007). Meta-analysis of stigma and mental health. *Soc. Sci. Med.* 65 245–261. 10.1016/J.SOCSCIMED.2007.03.015 17462800

[B51] Marcus B. Weigelt O. Hergert J. Gurt J. Gelléri P. (2017). The use of snowball sampling for multi source organizational research: some cause for concern. *Pers. Psychol.* 70 635–673. 10.1111/peps.12169

[B52] Morris S. B. (2012). Challenging the public stigma of mental illness: a meta-analysis of outcome studies. *Psychiatr. Serv.* 63 963–973. 10.1176/appi.ps.005292011 23032675

[B53] Muhidin S. Vizheh M. Moghadam Z. B. (2020). Anticipating COVID-19-related stigma in survivors and health-care workers: lessons from previous infectious diseases outbreaks – an integrative literature review. *Psychiatry Clin. Neurosci.* 74 617–618. 10.1111/pcn.13140 32889754

[B54] Murray D. R. Mark S. (2016). “The behavioral immune system: implications for social cognition, social interaction, and social influence,” in *Advances in Experimental Social Psychology*, Vol. 53 eds Olson J. M. Zanna M. P. (Amsterdam: Elsevier), 75–129.

[B55] Nguyen T. Croucher S. M. Diers-Lawson A. Maydell E. (2021). Who’s to blame for the spread of COVID-19 in New Zealand? Applying attribution theory to understand public stigma. *Commun. Res. Pract.* 7 379–396. 10.1080/22041451.2021.1958635

[B56] Nyblade L. Srinivasan K. Mazur A. Raj T. Patil D. S. Devadass D. (2018). HIV stigma reduction for health facility staff: development of a blended- learning intervention. *Front. Public Health* 6:165. 10.3389/fpubh.2018.00165 29977887 PMC6021510

[B57] Oaten M. Stevenson R. J. Case T. I. (2011). Disease avoidance as a functional basis for stigmatization. *Philos. Trans. R. Soc. B Biol. Sci.* 366 3433–3452. 10.1098/rstb.2011.0095 22042920 PMC3189356

[B58] Overholt L. Wohl D. A. Fischer W. A. Westreich D. Tozay S. Reeves E. (2018). Stigma and Ebola survivorship in Liberia: results from a longitudinal cohort study. *PLoS One* 13:e0206595. 10.1371/journal.pone.0206595 30485311 PMC6261413

[B59] Pappas G. Kiriaze I. J. Giannakis P. Falagas M. E. (2009). Psychosocial consequences of infectious diseases. *Clin. Microbiol. Infect.* 15 743–747. 10.1111/j.1469-0691.2009.02947.x 19754730 PMC7129378

[B60] Park J. H. Faulkner J. Schaller M. (2003). Evolved disease-avoidance processes and contemporary anti-social behavior: prejudicial attitudes and avoidance of people with physical disabilities. *J. Nonverbal Behav.* 27 65–87. 10.1016/j.obhdp.2009.08.002

[B61] Patel B. R. Khanpara B. G. Mehta P. I. Patel K. D. Marvania N. P. (2021). Evaluation of perceived social stigma and burnout, among health-care workers working in covid-19 designated hospital of India: a cross-sectional study. *Asian J. Soc. Health Behav.* 4 156–162. 10.4103/SHB.SHB_54_21

[B62] Payne B. K. Vuletich H. A. Lundberg K. B. (2017). The bias of crowds: how implicit bias bridges personal and systemic prejudice. *Psychol. Inq.* 28 233–248. 10.1080/1047840X.2017.1335568

[B63] Peirce J. W. (2007). PsychoPy-psychophysics software in python. *J. Neurosci. Methods* 162 8–13. 10.1016/j.jneumeth.2006.11.017 17254636 PMC2018741

[B64] Peng E. Y. C. Lee M. B. Tsai S. T. Yang C. C. Morisky D. E. Tsai L. T. (2010). Population-based post-crisis psychological distress: an example from the SARS outbreak in Taiwan. *J. Formos. Med. Assoc.* 109 524–532. 10.1016/S0929-6646(10)60087-3 20654792 PMC3106105

[B65] Person B. Sy F. Holton K. Govert B. Liang A. The NCID, SARS Community Outreach Team et al (2004). Fear and stigma: the epidemic within the SARS outbreak. *Emerg. Infect. Dis.* 10 358–363. 10.3201/eid1002.030750 15030713 PMC3322940

[B66] Phelan J. C. Link B. G. Dovidio J. F. (2008). Stigma and prejudice: one animal or two? *Soc. Sci. Med.* 67 358–367. 10.1016/j.socscimed.2008.03.022 18524444 PMC4007574

[B67] Pryor J. B. Reeder G. D. Yeadon C. Hesson-McInnis M. (2004). A dual-process model of reactions to perceived stigma. *J. Pers. Soc. Psychol.* 87 436–452. 10.1037/0022-3514.87.4.436 15491270

[B68] Pryora J. B. Reeder G. D. Wesselmann E. D. Williams K. D. Wirth J. H. (2013). The influence of social norms upon behavioral expressions of implicit and explicit weight-related stigma in an interactive game. *Yale J. Biol. Med.* 86 189–201. 23766740 PMC3670439

[B69] Ransing R. Ramalho R. de Filippis R. Ojeahere M. I. Karaliuniene R. Orsolini L. (2020). Infectious disease outbreak related stigma and discrimination during the COVID-19 pandemic: drivers, facilitators, manifestations, and outcomes across the world. *Brain Behav. Immun.* 89 555–558. 10.1016/j.bbi.2020.07.033 32731007 PMC7384410

[B70] Reinius M. Zeluf Andersson G. Svedhem V. Wettergren L. Wiklander M. Eriksson L. E. (2021). Stigma and Ebola survivorship in Liberia: results from a longitudinal cohort study. *PLoS One* 77:e0206595. 10.1371/journal.pone.0206595 30485311 PMC6261413

[B71] Rydell R. J. McConnell A. R. (2006). Understanding implicit and explicit attitude change: a systems of reasoning analysis. *J. Pers. Soc. Psychol.* 91 995–1008. 10.1037/0022-3514.91.6.995 17144760

[B72] Saeed F. Mihan R. Mousavi S. Z. Reniers R. L. E. P. Bateni F. S. Alikhani R. (2020). A narrative review of stigma related to infectious disease outbreaks: what can be learned in the face of the Covid-19 pandemic? *Front. Psychiatry* 11:565919. 10.3389/fpsyt.2020.565919 33343414 PMC7738431

[B73] Schaller M. Park J. H. (2011). The behavioral immune system (and why it matters). *Curr. Dir. Psychol. Sci.* 20 99–103. 10.1177/0963721411402596

[B74] Schmidt G. Weiner B. (1988). An attribution-affect-action theory of behavior: replications of judgments of help-giving. *Pers. Soc. Psychol. Bull.* 14 610–621. 10.1177/0146167288143021

[B75] Shamblaw A. L. Botha F. B. Dozois D. J. A. (2015). Accounting for differences in depression stigma between Canadian Asians and Europeans. *J. Cross Cult. Psychol.* 46 597–611. 10.1177/0022022115575076

[B76] Sotgiu G. Dobler C. C. (2020). Social stigma in the time of coronavirus disease 2019. *Eur. Respir. J.* 56 23–25. 10.1183/13993003.02461-2020 32631833 PMC7338401

[B77] Stangl A. L. Earnshaw V. A. Logie C. H. Van Brakel W. Simbayi L. C. Barré I. (2019). The health stigma and discrimination framework: a global, crosscutting framework to inform research, intervention development, and policy on health-related stigmas. *BMC Med.* 17:31. 10.1186/s12916-019-1271-3 30764826 PMC6376797

[B78] Stark T. H. Flache A. Veenstra R. (2013). Generalization of positive and negative attitudes toward individuals to outgroup attitudes. *Pers. Soc. Psychol. Bull.* 39 608–622. 10.1177/0146167213480890 23471320

[B79] Stevens F. Taber K. (2021). The neuroscience of empathy and compassion in pro-social behavior. *Neuropsychologia* 159:107925. 10.1016/j.neuropsychologia.2021.107925 34186105

[B80] Taylor S. Landry C. Rachor G. Paluszek M. Gordon J. Asmundson G. (2020). Fear and avoidance of healthcare workers: an important, under-recognized form of stigmatization during the COVID-19 pandemic. *J. Anxiety Disord.* 75:102289. 10.1016/j.janxdis.2020.102289 32853884 PMC7434636

[B81] Thornicroft G. Brohan E. Rose D. Sartorius N. Leese M. (2009). Global pattern of experienced and anticipated discrimination against people with schizophrenia: a cross-sectional survey. *Lancet* 373 408–415. 10.1016/S0140-6736(08)61817-6 19162314

[B82] Ugidos C. López-Gómez A. Castellanos M. Á Saiz J. González-Sanguino C. Ausín B. (2020). Evolution of intersectional perceived discrimination and internalized stigma during COVID-19 lockdown among the general population in Spain. *Int. J. Soc. Psychiatry* 68 55–63. 10.1177/0020764020975802 33274660 PMC8793305

[B83] van Bommel G. Thijs J. Miklikowska M. (2021). Parallel empathy and group attitudes in late childhood: the role of perceived peer group attitudes. *J. Soc. Psychol.* 161 337–350. 10.1080/00224545.2020.1840326 33138728

[B84] Vuletich H. A. Payne B. K. (2019). Stability and change in implicit bias. *Psychol. Sci.* 30 854–862. 10.1177/0956797619844270 31050916

[B85] Wang X. Huang X. Jackson T. Chen R. (2012). Components of implicit stigma against mental illness among Chinese students. *PLoS One* 7:e46016. 10.1371/journal.pone.0046016 23029366 PMC3461029

[B86] Weiner B. (1995). *Judgments of Responsibility: A Foundation for a Theory of Social Conduct.* New York, NY: Guilford Press.

[B87] Weiner B. (1996). Searching for order in social motivation. *Psychol. Inq.* 7, 199–216. 10.1207/s15327965pli0703_1

[B88] White Hughto J. M. Reisner S. L. Pachankis J. E. (2015). Transgender stigma and health: a critical review of stigma determinants, mechanisms, and interventions. *Soc. Sci. Med.* 147 222–231. 10.1016/j.socscimed.2015.11.010 26599625 PMC4689648

[B89] World Health Organization [WHO] (2020). *Social Stigma Associated with COVID-19 A Guide to Preventing and Addressing.* Geneva: WHO.

[B90] Wu T. H. Chang C. C. Chen C. Y. Wang J. D. Lin C. Y. (2015). Further psychometric evaluation of the self-stigma scale-short: measurement invariance across mental illness and gender. *PLoS One* 10:e0117592. 10.1371/JOURNAL.PONE.0117592 25659115 PMC4320062

[B91] Xin M. Luo S. She R. Yu Y. Wang S. Tao F. (2020). Negative cognitive and psychological correlates of mandatory quarantine during the initial COVID-19 outbreak in China. *Am. Psychol.* 75 607–617. 10.1037/amp0000692 32673008

[B92] Zhang M. Mu Y. Zhang Y. Kong Y. (2020). The effect of stigmatization on interpersonal interactions of stigmatized individuals. *Adv. Psychol. Sci.* 28 1564–1574. 10.3724/sp.j.1042.2020.01564

[B93] Zhao L. Wang Z. Guan J. Shen P. Zhao W. Zuo G. (2021). Coronavirus Disease 2019–Related Stigma in China: a descriptive study. *Front. Psychol.* 12:694988. 10.3389/fpsyg.2021.694988 34456809 PMC8385269

