# Supplementary material for: Comparisons Between COVID-19 Stigma and Other Stigmas: Distinct in Explicit Attitudes and Similar in Implicit Process
Source: Front Psychol. 2022 Apr 26;13:848993. doi: 10.3389/fpsyg.2022.848993 (PMC9087195; doi:10.3389/fpsyg.2022.848993)
Supplement: Supplementary file 1 [file Data_Sheet_1.docx]

**Supplementary Materials for**

Comparisons between COVID-19 stigma and other stigma: Distinct in explicit attitudes and similar in implicit process

**This file includes:**

Instruments and materials used in three studies

Table S1-S3

Fig. S1-S4

**Supplementary Materials**

**Questionnaire**

Negative attitude: Please evaluate the people below according to your real opinions.1 (very positive) to 7 (very negative).

Emotional reactions:

FEAR: How likely would you feel fear when you meet the following people? 1 (very unlikely) to 7 (very likely).

SYMPATHY: How likely would you feel sympathy when you meet the following people? 1 (very unlikely) to 7 (very likely).

Cognitive Processes:

ATTRUBUTION: How likely would you think the following people are caused by their own reasons? 1 (very unlikely) to 7 (very likely).

EMOTION REGULATION: How likely would you regulate your negative emotion when you meet the following people? 1 (very unlikely) to 7 (very likely).

Withdrawal/approach motivation:

AVOIDANCE: How likely would avoid the following people? 1 (very unlikely) to 7 (very likely).

HELPING: How likely would you help the following people? 1 (very unlikely) to 7 (very likely) .

Social interactions and evaluations:

BECOMING NEIGHBORS: How likely would you become neighbors with the following people ? 1 (very unlikely) to 7 (very likely).

TRUST: How likely would trust the following people ? 1 (very unlikely) to 7 (very likely).

DEVIATION OF SOCIAL NORMS: How likely would you think the following people have deviated from the social norms? 1 (very unlikely) to 7 (very likely).

SOCIAL HARMFULNESS: How likely would you think the following people have social harm? 1 (very unlikely) to 7 (very likely).

**Materials for the IAT experiment in Study 3.**

Positive adjectives: 可亲的，接近的，无传染性的，无危害的，无威胁的，正面的(Amiable, approachable, non-contagious, harmless, non-threatening, positive)

Negative adjectives: 可怕的，远离的，有传染性的，有危害的，有威胁的，负面的 (Scary, aloof, contagious, harmful, threatening, negative)

Nouns for the COVID-19 group: 新冠患者，新冠疑似，新冠病人，新冠确诊，新冠感染者，新冠携带者 (COVID-19 patients, COVID-19 probable patients, patients with the COVID-19, people who have been diagnosed with the COVID-19, people who suffered from the COVID-19, people carries the COVID-19 virus)

Nouns for the SARS group: 非典患者，非典疑似，非典病人，非典确诊，非典感染者，非典携带者 (SARS patients, SARS probable patients, patients with the SARS, people who have been diagnosed with the SARS, people who suffered from the SARS, people carries the SARS virus)

Nouns for the control group: 普通人，普通市民，普通群众，普通老百姓，社会成员，身体健康的 (Ordinary people, ordinary citizens, ordinary public, ordinary populace, members of society, healthy)

**Table S1. Negative attitudes towards the COVID-19 group and the SARS group in Study 1.**

|  |  |  | **Negative attitude score(M±SD)** | |  |  |
| --- | --- | --- | --- | --- | --- | --- |
| **Characteristic** | **N** | **%** | **COVID-19 group** | **SARS group** | **t** | ***p*** |
|  |  |  |  |  | t_COVID-19 = 1.92_ | 0.055 |
|  |  |  |  |  | t_SARS = 1.07_ | 0.292 |
| **Gender** |  |  |  |  |  |  |
| Male | 320 | 33.90% | 4.00±1.18 | 3.66±1.13 |  |  |
| Female | 624 | 66.10% | 3.84±1.26 | 3.57±1.18 |  |  |
| **Age(years)** |  |  |  |  |  |  |
|  |  |  |  |  | t_COVID-19 = -0.50_ | 0.621 |
|  |  |  |  |  | t_SARS = 1.14_ | 0.257 |
| <=30 | 377 | 39.89% | 3.87±1.17 | 3.65±1.07 |  |  |
| >30 | 568 | 60.11% | 3.90±1.28 | 3.57±1.21 |  |  |

***Note.*** The higher the negative attitude score, the more severe the stigma. T-test results show differences in COVID-19 and SARS stigma among subjects with different demographic characteristics (i.e., gender and age). Abbreviations: SD: standard deviation.

**Table S2. Post Hoc Comparisons of Disease type X Target people type in Study 1.**

| **Comparison** | | | |  |  |  |  |
| --- | --- | --- | --- | --- | --- | --- | --- |
| **disease** | **people** | **disease** | **people** | **Mean Difference** | **SE** | **t** | **p** |
| COVID-19 | patients | COVID-19 | recovered | 0.97 | 0.04 | 23.41*** | < .001 |
|  |  | SARS | patients | 0.57 | 0.04 | 15.35*** | < .001 |
|  |  | SARS | recovered | 0.98 | 0.04 | 22.47*** | < .001 |
|  | recovered | SARS | patients | -0.39 | 0.04 | -10.05*** | < .001 |
|  |  | SARS | recovered | 0.01 | 0.03 | 0.46 | 0.648 |
| SARS | patients | SARS | recovered | 0.41 | 0.03 | 12.96*** | < .001 |

***Note.*** df = 1086. **p*<0.05, ***p*<0.01, ****p*<0.001.

**Table S3. Negative attitudes towards the COVID-19 group and the SARS group in Study 2.**

|  |  |  | **Negative attitude score(M±SD)** | |  |  |
| --- | --- | --- | --- | --- | --- | --- |
| **Characteristic** | **N** | **%** | **COVID-19 group** | **SARS group** | **t** | ***p*** |
|  |  |  |  |  | t_COVID-19_ = -0.39 | 0.700 |
|  |  |  |  |  | t_SARS_ = 1.24 | 0.216 |
| **Gender** |  |  |  |  |  |  |
| Male | 107 | 38.35% | 4.01±1.17 | 4.03±1.04 |  |  |
| Female | 177 | 61.65% | 4.07±1.17 | 3.87±1.08 |  |  |
| **Age(years)** |  |  |  |  |  |  |
|  |  |  |  |  | t_COVID-19_ = -1.30 | 0.195 |
|  |  |  |  |  | t_SARS_ = 0.28 | 0.774 |
| <=30 | 251 | 89.96% | 4.02±1.16 | 3.93±1.08 |  |  |
| >30 | 28 | 10.04% | 4.32±1.23 | 3.87±0.99 |  |  |

***Note.*** The higher the negative attitude score, the more severe the stigma. T-test results show differences in COVID-19 and SARS stigma among subjects with different demographic characteristics (i.e., gender and age). Abbreviations: SD: standard deviation


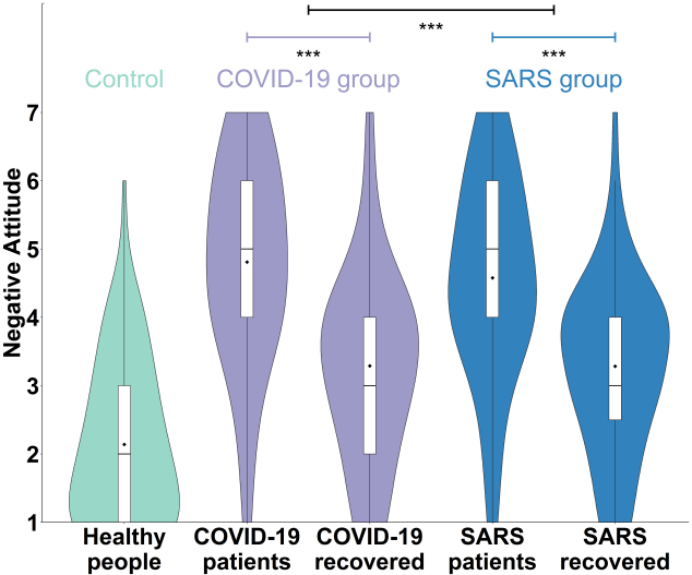


Figure.S1. Negative attitudes towards the COVID-19 group (i.e., the COVID-19 patients, people who recovered from COVID-19), the SARS group (i.e., the SARS patients and people who recovered from SARS), and the control group in Study 2. Higher scores indicate more negative attitudes. **p*<0.05, ***p*<0.01, ****p*<0.001.

**
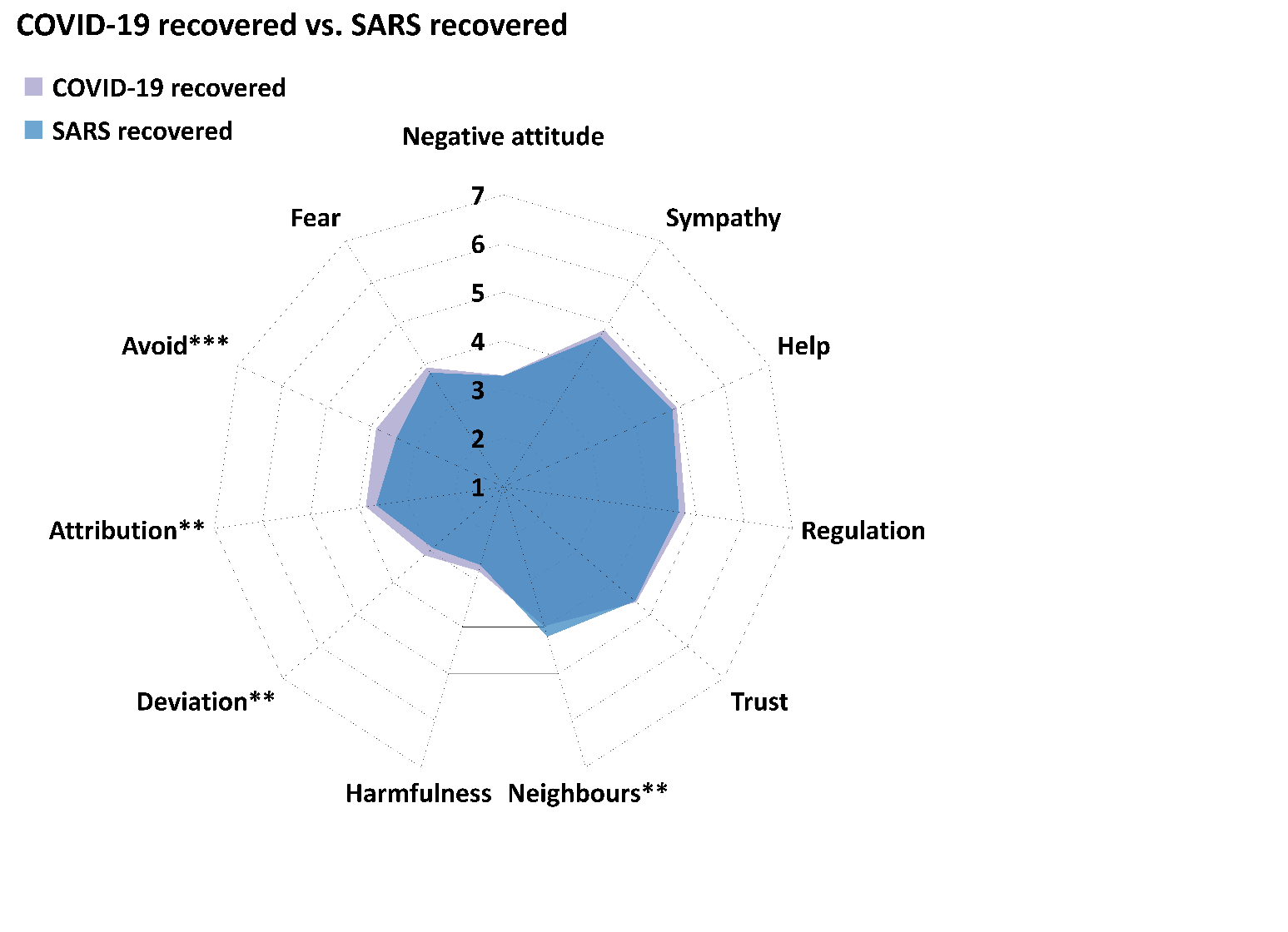
**

Figure. S2. Participants' explicit attitudes towards the people recovered from COVID-19 versus the people who recovered from SARS in terms of emotional (i.e., fear and sympathy), cognitive (i.e., attribution and regulation), motivational (i.e., avoid, help, and the willingness to become neighbors) and social dimensions (i.e., social harmfulness, social deviant and trust) in Study 2. Higher score indicates a higher level of possibility. **p*<0.05, ***p*<0.01, ****p*<0.001.


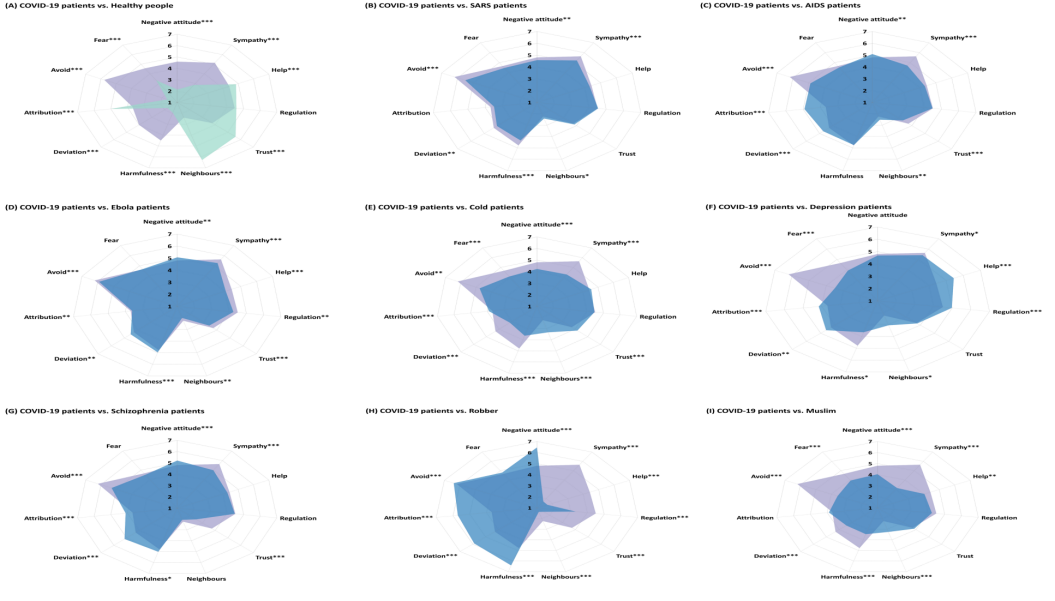


Figure. S3. The comparisons between participants' multiple attitudes towards the COVID-19 patients and other groups in Study 2. Higher score indicates a higher level of possibility. **p*<0.05, ***p*<0.01, ****p*<0.001.


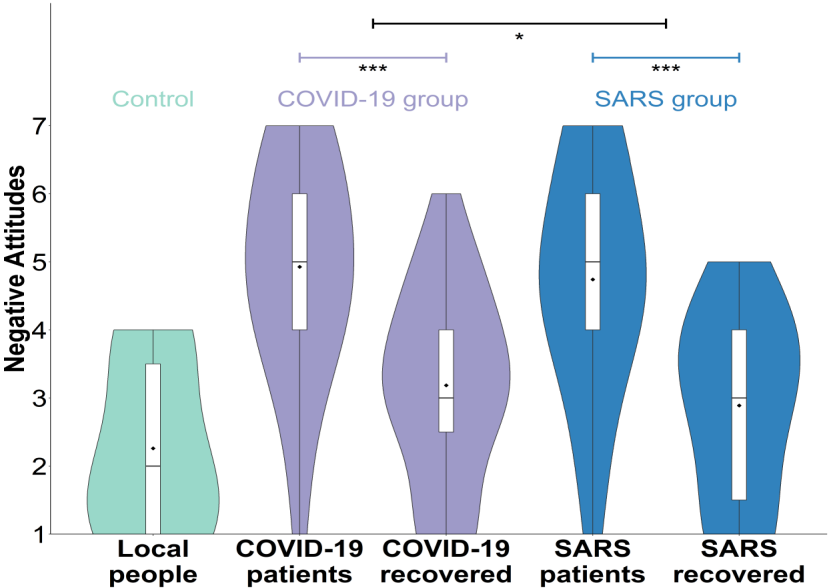


Figure. S4. Negative attitudes towards the COVID-19 group (i.e., the COVID-19 patients, people who recovered from COVID-19), the SARS group (i.e., the SARS patients and people who recovered from SARS), and the control group in Study 3. Higher scores indicate more negative attitudes. **p*<0.05, ***p*<0.01, ****p*<0.001.
